# Improvement in detecting cytomegalovirus drug resistance mutations in solid organ transplant recipients with suspected resistance using next generation sequencing

**DOI:** 10.1371/journal.pone.0219701

**Published:** 2019-07-18

**Authors:** Rubén López-Aladid, Alba Guiu, Maria Mar Mosquera, Francisco López-Medrano, Frederic Cofán, Laura Linares, Julián Torre-Cisneros, Elisa Vidal, Asunción Moreno, Jose María Aguado, Elisa Cordero, Cecilia Martin-Gandul, Jordi Carratalá, Nuria Sabé, Jordi Niubó, Carlos Cervera, Alicia Capón, Anna Cervilla, Marta Santos, Marta Bodro, Patricia Muñoz, Maria Carmen Fariñas, Andrés Antón, Maitane Aranzamendi, Miguel Montejo, Pilar Pérez-Romero, Oscar Len, Maria Ángeles Marcos

**Affiliations:** 1 Department of Clinical Microbiology, Hospital Clinic, Universidad de Barcelona, Institute for Global Health, (ISGlobal), Barcelona, Spain; 2 Unit of Infectious Diseases, Instituto de Investigación Hospital 12 Octubre (i + 12) University Hospital 12 de Octubre, Universidad Complutense, Madrid, Spain; 3 Department of Nephrology and Renal Transplant, Hospital Clinic, Universidad de Barcelona, Barcelona, Spain; 4 Department of Infectious Diseases, Hospital Clinic, Institut d'Investigacions Biomediques August Pi I Sunyer (IDIBAPS), Universidad de Barcelona, Barcelona, Spain; 5 Clinical Unit of Infectious Diseases, Hospital Universitario Reina Sofia-IMIBIC-UCO, Córdoba, Spain; 6 Department of Infectious Diseases, Instituto de Biomedicina de Sevilla (IBIS), University Hospital Virgen del Rocío, Sevilla, Spain; 7 Department of Infectious Diseases, Bellvitge University Hospital, IDIBELL, Barcelona, Spain; 8 Department of Clinical Microbiology, Bellvitge University Hospital, IDIBELL, Barcelona, Spain; 9 Department of Medicine, Division of Infectious Diseases, University of Alberta, Edmonton, Canada; 10 Department of Clinical Microbiology and Infectious Diseases, Hospital General Universitario Gregorio Marañón, Instituto de Investigación Sanitario Gregorio Marañón, Madrid, Spain; 11 Unit of Infectious Diseases, University Hospital Marqués de Valdecilla, Universidad de Cantabria, Santander, Spain; 12 Microbiology Service, Hospital Vall d'Hebron, Barcelona, Spain; 13 Microbiology Service, Hospital Universitario de Cruces, Bilbao, Spain; 14 Department of Infectious Diseases, Hospital Universitario de Cruces, Bilbao, Spain; 15 National Center for Microbiology, Instituto de Salud Carlos III, Majadahonda, Madrid, Spain; 16 Department of Infectious Diseases, Hospital Universitari Vall d'Hebrón, Uniiversidad Autónoma de Barcelona, Barcelona, Spain; University of Toledo, UNITED STATES

## Abstract

**Objetives:**

The aim of this study was to identify CMV drug resistance mutations (DRM) in solid organ transplant (SOT) recipients with suspected resistance comparing next-generation sequencing (NGS) with Sanger sequencing and assessing risk factors and the clinical impact of resistance.

**Methods:**

Using Sanger sequencing as the reference method, we prospectively assessed the ability of NGS to detect CMV DRM in the *UL97* and *UL54* genes in a nationwide observational study from September 2013 to August 2016.

**Results:**

Among 44 patients recruited, 14 DRM were detected by Sanger in 12 patients (27%) and 20 DRM were detected by NGS, in 16 (36%). NGS confirmed all the DRM detected by Sanger. The additional six mutations detected by NGS were present in <20% of the sequenced population, being located in the UL97 gene and conferring high-level resistance to ganciclovir. The presence of DRM by NGS was associated with lung transplantation (p = 0.050), the administration of prophylaxis (p = 0.039), a higher mean time between transplantation and suspicion of resistance (p = 0.038) and longer antiviral treatment duration before suspicion (p = 0.024). However, the latter was the only factor independently associated with the presence of DRM by NGS in the multivariate analysis (OR 2.24, 95% CI 1.03 to 4.87).

**Conclusions:**

NGS showed a higher yield than Sanger sequencing for detecting CMV resistance mutations in SOT recipients. The presence of DRM detected by NGS was independently associated with longer antiviral treatment.

## Introduction

Cytomegalovirus (CMV) infection is one of the most common complications in solid organ transplant (SOT) patients. Currently, prophylaxis and preemptive treatments have decreased the incidence of disease and death by CMV. However, mutations associated with resistance to antivirals have become an important problem with a high associated morbidity and mortality [1, 2].

In the transplant setting, the incidence of drug resistance mutations (DRM) is 5–12% depending on the group of patients studied but may be higher than 20% in patients with suspected DRM [3, 4].

Diagnostic laboratory testing should be performed whenever there is a criterion of suspicion of resistance since suboptimal response to treatment may have other causes, and inadequate empiric changes in therapy may have adverse effects [5, 6]. Genotypic testing by Sanger sequencing of the protein kinase gene (*UL97*) and DNA polymerase gene (*UL54*) is the usual method for detecting drug resistance and is the basis of the selection of alternative therapy [7]. However, Sanger DNA sequencing is unable to detect and quantify subpopulations of viral mutants of less than 20–30%, which may be crucial for selecting the most adequate therapy [8].

In the last years next generation sequencing (NGS) technologies have provided better understanding of the complexity of variant populations, increasing the detection of subpopulations of minority variants which have a prevalence of 1–3%. Nevertheless, to date, only a few studies have evaluated CMV DRM using NGS and most of these studies were carried out in a reduced number of patients [9– 12].

The present study was conducted within the context of the Group for the Study of Infection in Transplantation (GESITRA), constituting one of its major lines of clinical research. The nationwide network of Spanish hospitals participating in GESITRA has allowed the inclusion of a wide variety of SOT recipients.

The aim of this study was to identify CMV drug resistance mutations in solid organ transplant recipients with suspected resistance comparing NGS with Sanger sequencing and to assessing risk factors and the clinical impact of resistance detected by NGS.

## Materials and methods

### Settings and study population

We conducted a prospective observational study in nine hospitals included in the Spanish Network for Research in Infectious Diseases (REIPI). Adult SOT patients with suspected DRM were included in the study from September 2013 to August 2016. Resistance was suspected when there was persistent or increasing CMV viral load despite ≥ 2 weeks of appropriate antiviral treatment, or failure to have a significant improvement in clinical symptoms despite 2 weeks of full-dose antiviral therapy.[2,5,6]

The coordinating centre was the Hospital Clinic of Barcelona (Spain), which performed the genotypic resistance testing as well as the data analysis. Patient treatment and follow-up were conducted as per the protocol of each centre. All patients were initially treated with standard-dose ganciclovir/valganciclovir (GCV/VGCV) (adjusted to renal function).

Monitoring of CMV viral load was based on local quantitative PCR-based assays. On the suspicion of resistance, plasma samples were collected and frozen at -80°C in the respective hospital and sent in batches to the Microbiology Laboratory of the Hospital Clinic every 4 months. Thus, genotypic testing was not carried out in real time and all patients received empiric treatment on the presence of suspected drug resistance.

Data at patient inclusion and during follow-up were registered in a clinical database at each participating hospital. We reviewed medical histories in terms of viral response, rejection and mortality at 3 months after suspicion of resistance.

The DRM detected by Sanger sequencing and NGS were also included in the database.

CMV infection and disease and acute cellular rejection were defined as described previously [5, 6].

The study was endorsed by Group for the study of Infection in Transplantation (GESITRA) and approved by the Ethical Committee of Hospital Clínic (Comité Ético de Investigación del Hospital Clínic de Barcelona, CEIC), approval number HCB/2012/7598) as the reference committee for the all participating centres according to legal regulations in Spain.

### Microbiological studies

In the coordinating laboratory, all samples were kept at -80°C until processing. DNA extraction was performed in 500 μL of each plasma sample using the QIAsymphony system (Qiagen, Hilden, Germany). CMV viral load was confirmed by CMV Real Time PCR (Nanogen Advanced Diagnostics, Italy) according to the manufacturer’s instructions.

Subsequently, all samples were analysed by Sanger sequencing based on PCR amplification of the CMV *UL97* protein kinase gene (codons 400–670) in a single fragment and the *UL54* DNA polymerase gene (codons 300–1000) in four fragments [3].

Afterwards, antiviral resistance NGS, using Ion Torrent PGM (Ion Personal Genome Machine, Ion Torrent Life Technologies, South San Francisco, CA) was performed by flanking both the *UL97* and *UL54* gene.

### Primer and library design for NGS

To create DNA library, *UL97* and *UL54* primers and PCR conditions previously validated and described by Sahoo MK et al were used [10].

After establishing the whole technique, pilot tests were performed in Ion Torrent PGM in order to adjust the reproducibility of the technique. The test of reproducibility was carried out in a total of 6 control samples which included: 2 samples without suspicion of resistance, 2 samples from QCMD with known DRMs, and 2 clinical samples with known DRMs tested by Sanger. The results were the same in two sequencing experiments. Each run had a pool of the 6 samples in 3 different libraries with the same DNA extract. [13]

The limit of detection to avoid false positive results was defined as 5% according to Sahoo et al [10]

### Sequencing and bioinformatic analyses

Forty-eight samples were sequenced in the Ion PGMTM 35 System for NGS. Quality assessment of the FASTQ files was performed using FastQC–version 0.11.3 [14]. The resulting reads were aligned to the human herpesvirus 5 strain Merlin reference genome (GenBank: AY446894.2) using the BWA-MEM algorithm from BWA version 0.7.12 with default parameters [15]. Alignments were sorted and indexed using Sambamba version 0.5.1 [16]. Duplicate reads in sorted BAM files were identified and removed using Picard tools [17]. GATK base quality score recalibration, insertion and delection (INDEL) realignment, and duplicate removal were applied [18]. The discovery of single nucleotide polymorphisms (SNP) and INDEL and genotyping were simultaneously performed across 12 samples to optimise this method using standard filtering and variant quality score recalibration according to the GATK Best Practices recommendations [19]. Genetic variant annotation and prediction of the effects of variants on genes (such as amino acid changes) were done using snpEff [20]. To do this a snpEff database was created using the information of the reference genome provided by GenBank. The resulting variant call format (VCF) files were post-processed with in-house R [21] scripts using Bioconductor packages [22] to identify mutations by gene (i.e. *UL54* and *UL97*) in order to find the relative position of each mutation to the first position of the corresponding gene and to filter mutations by missing calls by depth at a sample level (DP> = 5), by allele depth (AD> = 5), and by poor quality calls (in which the percentile of minimum quality accepted was 5%).

An average 247382 raw sequences per sample were obtained in the samples sequenced in this study. After pipeline passing quality filters 142.078 reads were obtained per sample.The mean coverage among the different amplicons was up to 2000X

### Statistical analysis

A database was developed for the registry of the patients enrolled in Open Clinica 3.1 [GNULesser General Public License (GNU LGPL)].

Categorical variables are expressed as number and percentage of patients, and the median (interquartile range [IQR]) is used for continuous variables with a non-normal distribution and the mean (standard deviation [SD]) for those with a normal distribution. Categorical variables were compared using the X^2^ test or the Fisher exact test. Continuous variables were compared using the *t* test or the non-parametric Mann-Whitney test. Logistic regression analyses were performed to identify variables associated with the presence of mutations. Variables showing a significant result in the univariate analyses (p<0.05) were included in the multivariate model (forward stepwise procedure). The Hosmer-Lemeshow goodness-of-fit test was performed to assess the overall fit of the models [23]. The area under the receiver operating characteristic (ROC) curve of the multivariate model to predict the presence of mutations was calculated. Internal validation of the prediction model was conducted using ordinary nonparametric bootstrapping with 1,000 bootstrap samples and bias-corrected, accelerated 95% CIs [24] (S1 Text; S1 Table). The level of significance was set at 0.05 (2-tailed). All analyses were performed using IBM SPSS Statistics 22.0 (Armonk, New York, USA).

### Results

During the study period, 48 adults who had undergone SOT and in whom CMV antiviral resistance was suspected were included in the study. Four samples with a low CMV viral load (< 1.000 copies/ml) were excluded from the analysis because neither Sanger sequencing nor NGS could be carried out. Therefore, 44 patients were finally included. The baseline characteristics of the study population are shown in Table 1.

**Table 1:** Comparison of the clinical characteristics of the study population at baseline.

Variables	Overall	Without Mutations	With Mutations	P value
	(n = 44)	(n = 28)	(n = 16)	
**Age, mean (SD), years**	52.5 (12.3)	54.1 (11.8)	49.8 (12.9)	0.26
**Male sex, n (%)**	32 (72.7)	22 (78.6)	10 (62.5)	0.30
**Type of transplant, n (%)**				0.24
Kidney	19 (43.2)	13 (46.4)	6 (37.5)	0.57
Liver	9 (20.5)	6 (21.4)	3 (18.8)	>0·99
Heart	8 (18.2)	5 (17.9)	3 (18.8)	>0.99
Lung	5 (11.4)	1 (3.6)	4 (25.0)	0.050
Kidney-pancreas	3 (6.8)	3 (10.7)	0 (0)	0.29
**CMV serostatus, n (%)**^**a**^				0.21
D+/R-	26 (61.9)	14 (53.8)	12 (75.0)	0.17
D+/R+	13 (31.0)	10 (38.5)	3 (18.8)	0.30
D-/R+	2 (4.8)	2 (7.7)	0 (0)	0.52
D-/R-	1 (2.4)	0 (0)	1 (6.3)	0.38
**Induction therapy, n (%)**				
Basiliximab	21 (47.7)	16 (57.1)	5 (31.3)	0.098
Antithymocyte globulin	10 (22.7)	7 (25.0)	3 (18.8)	0.72
**Initial maintenance therapy, n (%)**^**b**^				
Mycophenolate mofetil	36 (81.8)	23 (82.1)	13 (81.3)	>0.99
Tacrolimus	35 (79.5)	24 (85.7)	11 (68.8)	0.25
Steroids	34 (77.3)	21 (75.0)	13 (81.3)	0.72
Cyclosporine	6 (13.6)	2 (7.1)	4 (25.0)	0.17
mTOR inhibitors	5 (11.4)	2 (7.1)	3 (18.8)	0.34
**CMV prophylaxis, n (%)**	24 (54.5)	12 (42.9)	12 (75.0)	0.039
≤3 months^c^	18 (75.0)	10 (83.3)	8 (66.7)	0.64
≥6 months^c^	6 (25.0)	2 (16.7)	4 (33.3)	

Abbreviations: D-, negative donor; D+, positive donor; R-, negative recipient; R+, positive recipient; mTOR, mammalian target of rapamycin; n, number of cases; SD, standard deviation

^a^ CMV serostatus not specified in 2 patients

^b^ May have had more than 1 initial maintenance

^c^ Percentages calculated for patients with CMV prophylaxis

Based on sequence analysis, 12 out of 44 (27%) patients showed CMV DRM by Sanger sequencing, while 16 patients (36%) showed mutations by NGS (Table 2). All the 16 patients had mutations in the UL97 gene and in addition, two patients had one mutation each in the UL54 gene (D413N and P522A). The NGS identified six low abundance resistance mutations that had not been reported by Sanger sequencing, since they were presented in <20% of the viral population. Patients 8 and 16 showed two mutations in UL97 (C592G + M460V and L595W + A594V respectively); in both cases. In these two patients, M460V and A594V, respectively, were only detected by NGS. These six mutations were reproducibly detected at similar levels in two independent sequencing experiments. All the DRM identified by Sanger were also identified by NGS. The lowest depth coverage obtained related to a resistance mutation was 7% of the viral subpopulations.

**Table 2:** Detection of cytomegalovirus drug resistance mutations in the UL97 and UL54 genes by Sanger and next-generation sequencing.

PATIENT	SANGER *UL97*	NGS (Coverage %) *UL97*	GCV^a^ RATIO	SANGER *UL54*	NGS Coverage % *UL54*	GCV/FOS/CDV^a^ RATIO
1		L595S (15%)	9.2			
2		M460V (19%)	8.3			
3	A594V	A594V (20%)	8.3			
4	M460V	M460V (29%)	8.3			
5		M460V (19%)	8.3			
6		L595S (14%)	9.2			
7	L595S	L595S (20%)	9.2			
8	C592G	C592G+M460V (26%/14%)	2.9/8.3			
9	H520Q	H520Q (30%)	10			
10	M460V	M460V (31%)	8.3	P522A	P522A (22%)	3/1/4.1
11	L595S	L595S (20%)	9.2			
12	M460V	M460V (26%)	8.3			
13	M460I	M460I (31%)	5	D413A	D413A (24%)	6.5/0.8/11
14	A594P	A594P (31%)	3			
15	L595S	L595S (23%)	9.2			
16	L595W	L595W+A594V (21%/7%)	5.1/8.3			

^a^IC_50_ of mutant /IC_50_ of wild type. If IC_50_ >5 the resistance was considered of high grade while an IC_50_ <3 was considered as low-grade resistance.

GCV: ganciclovir, FOS: foscarnet, CDV:cidofovir

The detection of mutations by NGS allowed the differentiation and comparison of the patients with and without mutations (Tables 1 and 3). The presence of mutations was more frequent in lung transplant recipients (p = 0.050) and in patients receiving prophylaxis (42.9% *vs*. 75%, p = 0.039), regardless of its duration. The median time between transplantation and suspicion of resistance was significantly longer in the group with DRM (211.5 days [87.5; 285.0] vs 108.5 days [43.5; 201.5] p = 0.038) as was the median treatment duration before suspicion of resistance (41.5 days [18.0; 75.5] vs 16.0 days [14.5; 22.5] (p = 0.024). At the time of suspicion of resistance, 75% of patients without mutations were receiving therapy for asymptomatic CMV replication and 50% of patients with DRM were receiving treatment for CMV disease. CMV disease was diagnosed in 54.5% of patients. However, biopsy confirmation was achieved in only 3 patients. All the patients were receiving standard GCV/VGCV doses at the time of suspicion of resistance and in the absence of DRM study results the treatment was empirically modified accordingly. In patients with a DRM, mTORi was added or changed empirically (50%, p = 0.009). No statistically significant differences were observed between the patients with and without mutations in relation to outcome at 3 months (Table 3).

**Table 3:** Data at the onset and beyond the suspicion of resistance.

Variables	Overall	Without Mutations	With Mutations	P value
	(n = 44)	(n = 28)	(n = 16)	
**Time interval between transplantation and suspicion, median (IQR), days**	116.5 (60.0; 232.5)	108.5 (43.5; 201.5)	211.5 (87.5; 285.0)	0.038
**Days of therapy before suspicion, median (IQR)**	20.0 (15.0; 48.0)	16.0 (14.5; 22.5)	41.5 (18.0;75.5)	0.024
**Treatment at the time of suspicion, n (%)**				
Preemptive treatment	28 (63.5)	21 (75.0)	7 (43.8)	0.038
Disease treatment	15 (34.1)	7 (25.0)	8 (50.0)	0.092
Prophylaxis	1 (2.3)	0 (0)	1 (6.3)	0.36
**CMV viral load at the time of suspicion, median (IQR), copies/ml**	11,304 (3,521; 64,105)	7,088 (2,415; 47,555)	36,236 (9,609; 65,247)	0.17
**Symptoms at the time of suspicion, n (%)**^**a**^	24 (54.5)	14 (50)	10 (62)	0.53
Viral syndrome^b^	13 (29.5)	10 (35.7)	3 (18.8)	0.31
Gastrointestinal disease^c^	8 (18.2)	3 (10.7)	5 (31.3)	0.12
Pneumonitis	4 (9.1)	2 (7.1)	2 (12.5)	0.61
Retinitis	2 (4.5)	2 (7.1)	0 (0)	0.53
Hepatitis	1 (2.3)	1 (3.6)	0 (0)	>0.99
Disseminated (>1 end organ)	1 (2.3)	0 (0)	1 (6.3)	0.36
**Therapeutic strategy on suspicion, n (%)**^**d**^				
Increase GCV/VGC dose	15 (34.1)	10 (35.7)	5 (31.3)	0.764
Continue with standard GCV/VGC^e^ dose	18 (40.9)	13 (46.4)	5 (31.3)	0.325
Decreased immunosuppression	12 (27.3)	6 (21.4)	6 37.5)	0.303
Switch or add mTOR inhibitors	11 (25.0)	3 (10.7)	8 (50.0)	0.009
Switch or add foscanet	8 (18.2)	3 (10.7)	5 (31.3)	0.12
**Outcome (at 3 months)**				
Viral response				
Total suppression of CMV replication	25 (56.8)	18 (64.3)	7 (43.8)	0.186
Incomplete suppression	19 (43.2)	10 (35.7)	9 (56.3)	0.156
**Final outcome**^**f**^				
Graft rejection	5 (11.9)	3 (11.1)	2 (13.3)	>0.99
Death	5 (11.9)	3 (11.1)	2 (13.3)	>0.99

Abbreviations: IQR, interquartile range; mTOR, mammalian target of rapamycin; n, number of case

^a^ May have had more than 1 symptom at the time of suspicion

^b^ Viral syndrome: fever >38°C(for at least 2 days in a 4 day period) associated with leucopenia, thrombocytopenia or increase in transaminases

^c^ Gastrointestinal disease include: colitis, gastritis

^d^ May have had more than 1 therapeutic strategy on suspicion

^e^ Continue with standard prophylactic or treatment dose of GCV/VGC

^f^Final outcome not specified in 2 patients

Table 4 shows the clinical and virological data of the SOT recipients with known CMV DRM. Of the variables associated with the presence of resistance mutations in the univariate logistic regression analyses (S2 Text. S2 Table), treatment duration before suspicion of resistance was the only factor independently associated with the presence of mutations in the multivariate analysis (log-transformed scale, OR 2.24, 95% CI 1.03 to 4.87). The area under the ROC curve was 0.71 (95% CI 0.53 to 0.88) for the final model of mutations (S1 Fig).

**Table 4:** Clinical and virological data in solid organ transplant recipients with cytomegalovirus resistance mutations.

	Type of Transplant (CMV serostatus)	Induction therapy	Prophylaxis (months)	Treatment on suspicion of resistance^a^ (Days)	Symptomatic on suspicion of resistance	*UL97/UL54* DRM (Days post-TX)	CMV viral ^b^ (copies/ml)	Therapeutic adaptation on suspicion of resistance	Virological and/or clinical outcome ^c^
1	Kidney D+/R-	ATG	3 M	Disease VGC 900mg/12h (16)	Suspected colitis	L595S (91)	4.180	GCV/VGC increased mTORi	IS.
2	Kidney D+/R+	Basiliximab	No	Preemptive treatment VGC 450mg/12h (15)	No	M460V (68)	55.253	GCV/VGC increased	CS.Total resolution
3	Liver D+/R-	No	No	Preemptive treatment VGC 450mg/12h(15)	No	A594V 63)	9.300	GCV/VGC increased ISP decreased	IS. Cell-mediated rejection
4	Liver D+/R-	No	3M	Disease VGC 450mg/12h(15)	Viral syndrome^d^, Suspected colitis	M460V (203)	3.849	ISP decreased Continue with GCV/VGCV	CS.Total resolution
5	Kidney D+/R+	Basiliximab	1M	Disease VGC 450mg/12h(84)	Suspected gastritis	M460V (235)	11.234	ISP decreased GCV/VGC increased mTORi	CS. Total resolution
6	Kidney D+/R+	ATG	No	Disease VGC 450mg/12h(67)	Viral syndrome ^d^ Suspected colitis	L595S (58)	114.167	GCV/VGC increased	CS.Total resolution
7	Heart D+/R-	No	3M	Disease VGC 100mg/12h (20)	Viral syndrome ^d^	L595S (350)	154.486	GCV/VGC increased	IS
8	Kidney D+/R-	ATG	3M	Disease VGC 450mg/12h(61)	Suspected colitis	C592G+M460V (102)	11.605	GCV/VGC increased. mTORi	IS
9	Lung D+/R-	No	12M	Prophylaxis VGC 900mg/24h(38)	Confirmed pneumonitis	H520Q (210)	33.772	ISP Decreased. FOS. mTORi	CS.Total resolution
10	Lung D+/R-	No	12M	Preemptive treatment VGC 900mg/12h(96)	No	M460V/P522A (335)	68.494	ISP Decreased .FOS. mTORi	IS
11	Lung D+/R-	No	6M	Preemptive treatment VGC 900mg/12h(26)	No	L595S (210)	38.700	ISP Decreased .FOS. mTORi	CS.Total resolution
12	Liver D+/R-	No	No	Disease VGC 450mg/12h (45)	Confirmed pneumonitis and retinitis	M460V (340)	9.919	Continue with GCV/VGCV. mTORi	IS. Death
13	Lung D+/R-	Basiliximab	12M	Preemptive treatment VGC 450mg/12h(231)	No	M460I/D413N (400)	62.000	GCV/VGC increased. Leflunomide	IS. Cell-mediated rejection
14	Heart D+/R-	Basiliximab	1M	Preemptive treatment VGC 450mg/12h(113)	Viral syndrome^d^	A594P (331)	59.879	Continue with GCV/VGCV. Maribavir	IS
15	Kidney D+/R-	No	1M	Disease VGC 450mg/12h(28)	Viral syndrome^c^ Suspected colitis	L595S (112)	721.000	Switch to FOS	IS. Death
16	Heart D+/R-	Basiliximab	2M	Preemptive treatment VGC 900mg/12h(51)	Viral syndrome^d^	L595W+A594V (84)	4.120	GCV/VGC increased. FOS. mTORi	CS.Total resolution

CMV, cytomegalovirus; D, donor; R, recipient; TX, transplantation; GCV, ganciclovir; VGC, valganciclovir; FOS, foscarnet, CDV, cidofovir; ATG, antithymocyte globulin; ISP, immunosuppression; IS, incomplete suppression; CS, total supression of CMV replication

^a^Drug/doses

^b^CMV viral load on suspicion of resistance

^c^Virological and clinical outcomes at 3 months

^d^Presence of fever >38°C (for at least 2 days in a 4 day period) associated with leucopenia, thrombocytopenia or an increase in transaminases

## Discussion

Drug-resistant CMV infection is an important emerging problem in SOT recipients. However, it may be underdiagnosed since mutation analysis is not routinely performed and the current gold-standard Sanger sequencing method lacks sufficient sensitivity [1]. In the present study, a NGS was compared with Sanger sequencing to identify mutations associated with resistance in SOT patients with suspicion of resistance. Among the 44 patients recruited, 14 DRM were detected by Sanger in 12 patients (27%) and 20 DRM were detected with NGS in 16 patients (36%), respectively. NGS showed a higher sensitivity for the detection of mutations present in <20% of the population sequenced which were not found by Sanger sequencing. Univariate analysis of the clinical data revealed that the presence of DRM detected by NGS was associated with lung transplantation (p = 0.050), prophylactic treatment (p = 0.039), a higher mean time between transplantation and suspicion of resistance (p = 0.038) and longer treatment duration with GCV or VGCV before suspicion (p = 0.024). However, the latter was the only factor independently associated with the presence of resistance mutations in the multivariate analysis.

Several aspects differentiate the present stydy from other similar studies in the field reported to date, and these aspects can be summarized as follows:NGS was addressed to the *UL97* and *UL54* genes, a relatively high number of patients, undergoing different solid organ transplanted were studied, and associated risk factors were also evaluated.

The current gold standard for the genotypic detection of CMV drug resistance is Sanger sequencing of the *UL97* and *UL54* genes. However, this technique cannot detect viral variants that represent < 20% of the viral population [9, 10]. This lack of sensitivity can lead to continued administration of an antiviral to which resistance has developed and the subsequent expansion of initially minority populations of resistant variants leading to the development of treatment failure, increased morbidity and shortened graft survival. On the other hand, modifications in therapy following the identification of GCV resistance is associated with more rapid viral clearance, further emphasising the importance of timely detection of resistance [7, 11, 12]. Recent studies have shown that NGS methods can provide new insights into viral diversity and in the detection of low-abundance variants by the analysis of thousands of amplicons in a single experiment [10, 25]. However, so far, only a few studies have focused on CMV-resistant mutants. Most studies have included few patients and only some cover the UL97 gene [11, 12]. An exception to this is the study by Sahoo et al which evaluated DRM in both of the genes involved using NGS in a considerable number of specimens [10]. However, this study did not include an analysis of clinical factors. In our study, NGS identified six low abundance resistant mutations that had not been reported by Sanger sequencing, thus suggesting that NGS is more sensitive than Sanger since the use of high throughput sequencing allowed the detection of viral subpopulations with a prevalence of 7%. All six mutations were located in the *UL97* gene and showed high-level resistance to GCV [26, 27]. In addition, two of these six DRM identified were present as mixed mutations in two patients which may have important implications in patient management. Therefore, studies with prospective monitoring of the abundance of such mutations over time and an assessment of their association with virologic failure are necessary. *UL54* DNA polymerase mutations are typically selected after prolonged GCV exposure and were revealed in 2 lung transplant patients who had received a longer prophylaxis and preemptive treatment [28, 29]. However, compared to Sanger sequencing NGS did not improve the detection sensitivity of DRM in the *UL54* gene in concordance with the results of Sahoo et al [10].

In the present study longer treatment duration with antivirals before suspicion of resistance was identified as a risk factor associated with DRM (p = 0.24)[1, 2], and is considered a major factor in the selection and emergence of resistance. Lung transplantation showed the highest incidence of DRM. It is important to note that 4 out of 5 lung transplant patients in our study presented DRM and all had received lengthy preemptive treatment and antiviral prophylaxis [29, 30]. Moreover, the presence of DRM was more frequent among transplant recipients who had received prophylaxis (p = 0.039). Nonetheless, other factors may have also contributed to this finding. The interdependence of host, virus and antiviral therapy is likely associated with the development of DRM [31]. In fact, all the patients included in this study had one or more known risk factors predisposing to DRM [1, 2]. Therefore, according to current consensus guidelines genotypic testing confirmation should be done irrespective of any exclusion criteria whenever antiviral resistance is suspected [5, 6], since resistance might otherwise be underestimated similar to what has been described in previous reports [32]. In the present study, DRM were found in unusual situations, such as during prophylaxis in one patient and in 3 patients with CMV D+/R+ serostatus.

Previous studies have reported antiviral resistance to be associated with increased attributable morbidity and mortality [2]. This was not found in the present study, likely because patients were only clinically followed during the first three months. Moreover, clinicians were unaware of the presence of DRM in real time, and therefore, treatment was empirically modified. Thus, we were unable to evaluate the outcome of the patients according to Sanger or NGS sequencing results. This should be addressed in future studies.

In our cohort, 64% of the patients suspected of having resistance to antivirals did not present DRM. Therefore, as suggested previously other factors might contribute to treatment failure [33]. Emphasis should be given to prioritizing the optimization of host factors rather than switching antiviral medications, thereby adjusting immunosuppressive therapy whenever possible. Additionally, blood GCV levels were not available and were not performed during the clinical care of the patients studied, and thus, sub-therapeutic GCV levels may be a possible cause of non-response to treatment in some patients. On the other hand, a recent study of serial specimens received for genotypic testing showed that the first specimen tested negative for DRM in more than half of the patients, although about two-thirds eventually became positive on repeated testing [4]. It could therefore be suggested that patients without DRM but with suspected criteria of resistance should be closely monitored and new samples should be studied for viral mutations.

Our study has limitations regarding the modest sample size and the lack of balance among groups although it does reflect real life in a nation-wide cohort study. Our results should be confirmed in larger and better balanced cohorts. Another limitation is that the analysis of GCV resistance of the mutations was performed only when there was suspicion; Nonetheless, these patients represented the group with the highest risk for antiviral resistant CMV.

In summary, the development of DRM may be a direct effect of a longer duration of antiviral treatment, and thus, the implementation of effective CMV prevention strategies that minimise drug exposure could lead to lower resistance rates. Although the clinical risk factors for drug resistance have been relatively well defined, genotypic testing should be performed in all patients with suspected criteria of resistance. Despite the lack of standardized protocols and the complexity of the bioinformatics analysis for the use of NGS in routine clinical practice, the results of this study suggests that NGS technology improves the genotypic diagnosis of DRM, fundamentally in low-abundance variants and mixed populations allowing early detection of the emergence of CMV-resistant strains and subsequent targeted adjustment of therapy.

## Supporting information

S1 Text Methods of statistical analysis. (DOC)

S2 Text Results of statistical analysis. (DOC)

S3 Text References of statistical analysis. (DOC)

S1 Table Internal validation of prediction model for mutations using nonparametric bootstrap technique. (DOC)

S2 Table Significant univariate and multivariable logistic regression analyses for mutations. (DOC)

S1 Fig Receiver operating characteristic curve analysis of significant variables derived from the logistic regression model for the ability to predict mutations. (DOC)
