# Supplementary material for: Improvement in detecting cytomegalovirus drug resistance mutations in solid organ transplant recipients with suspected resistance using next generation sequencing
Source: PLoS One. 2019 Jul 18;14(7):e0219701. doi: 10.1371/journal.pone.0219701 (PMC6638921; doi:10.1371/journal.pone.0219701)
Supplement: S1 Text — (DOC) [file pone.0219701.s001.doc]

**S1 Text.** **Methods of statistical analysis**

To identify factors associated with the presence of mutation a logistic regression model was used. Variables were included in the multivariate model when univariate comparisons yielded a level of significance of p<0.15. The following variables were tested: age, sex, CMV pre-transplant donor/recipient serology (D/R), type of transplant, prophylaxis and induction and maintenance therapy received, number of days after transplantation, if the patient was receiving prophylaxis, preemptive or disease treatment, viral load, CMV disease, and the dose and number of days of GCV or VGCV received prior to the suspicion. Finally, a forward stepwise selection (p_in_<0.05, p_out_>0.10) was used to determine factors associated to the presence of mutations. The odds ratio (OR) and 95% confidence interval (CI) were calculated. For continuous variables with non-normal distribution a logarithmic transformation was used. To identify the problem of collinearity, the r coefficient of two variables were calculated. When two independent variables were highly correlated (*r* >|±0.30|), the variable with the largest variance was excluded from the multivariate analysis [1]. The Hosmer-Lemeshow goodness-of-fit test was performed to assess the overall fit of the models [2]. Internal validation of the prediction model was conducted using ordinary nonparametric bootstrapping with 1,000 bootstrap samples and bias-corrected (S1Table A), accelerated 95% confidence intervals (CIs) [3]. The area under the receiver operating characteristic (ROC) curve was constructed for the ability to predict patients with mutations, using variables derived from the multivariate logistic regression model.
