# Supplementary material for: Improvement in detecting cytomegalovirus drug resistance mutations in solid organ transplant recipients with suspected resistance using next generation sequencing
Source: PLoS One. 2019 Jul 18;14(7):e0219701. doi: 10.1371/journal.pone.0219701 (PMC6638921; doi:10.1371/journal.pone.0219701)
Supplement: S2 Text — (DOC) [file pone.0219701.s002.doc]

**S2 Text. Results of statistical analysis**

Internal validation of the logistic regression model was conducted using bootstrapping with 1,000 samples and demonstrated robust results with small 95% CI around the original coefficient (S2Table) (S1 Fig.).
