# Supplementary material for: Improvement in detecting cytomegalovirus drug resistance mutations in solid organ transplant recipients with suspected resistance using next generation sequencing
Source: PLoS One. 2019 Jul 18;14(7):e0219701. doi: 10.1371/journal.pone.0219701 (PMC6638921; doi:10.1371/journal.pone.0219701)
Supplement: S3 Text — (DOC) [file pone.0219701.s003.doc]

**S3 Text. References of statistical analysis**

1.- Healey JF. Statistics: a tool for social research. Belmont, CA: Wadsworth Publishing Company,2016.

2.- Hosmer D, Lemeshow S. Applied logistic regression. New York: Wiley, 1989.

3.- Efron B, Tibshirani R. An introduction to the bootstrap (Monographs on statistics and applied probability. New York: Chapman and Hall, 1993.
