# Supplementary material for: Improvement in detecting cytomegalovirus drug resistance mutations in solid organ transplant recipients with suspected resistance using next generation sequencing
Source: PLoS One. 2019 Jul 18;14(7):e0219701. doi: 10.1371/journal.pone.0219701 (PMC6638921; doi:10.1371/journal.pone.0219701)
Supplement: S1 Table — (DOC) [file pone.0219701.s004.doc]

**S1 Table. Internal validation of prediction model for mutations using nonparametric bootstrap technique**

| **Variable** | **Original** | **Bias** | **SE** | **P value** | **95% BCa CI** |
| --- | --- | --- | --- | --- | --- |
| Log treatment duration before suspicion of resistance | 0.806 | 0.171 | 0.662 | 0.072 | 0.111 to 2.487 |

Abbreviations: BCa indicates adjusted bootstrap; CI, confidence interval; SE, standard error.
