# Supplementary material for: Improvement in detecting cytomegalovirus drug resistance mutations in solid organ transplant recipients with suspected resistance using next generation sequencing
Source: PLoS One. 2019 Jul 18;14(7):e0219701. doi: 10.1371/journal.pone.0219701 (PMC6638921; doi:10.1371/journal.pone.0219701)
Supplement: S2 Table — (DOC) [file pone.0219701.s005.doc]

**S2 Table. Significant univariate and multivariable logistic regression analyses for mutations**

| **Variable** | **Univariate**^a^ | | | **Multivariable^bc^** | | |
| --- | --- | --- | --- | --- | --- | --- |
|  | **OR** | **95% CI** | **P-value** | **OR** | **95% CI** | **P-value** |
| **Type of transplant: lung** | 9.00 | 0.91 to 89.27 | 0.061 | - | - | - |
| **Induction therapy: basiliximab** | 0.34 | 0.09 to 1.24 | 0.10 | - | - | - |
| **Initial maintance therapy: cyclosporine** | 4.33 | 0.70 to 27.01 | 0.12 | - | - | - |
| **CMV prophylaxis^d^** |  |  | 0.093 | - | - | - |
| No | 1.00 | - | - | - | - | - |
| 1-3 months | 3.20 | 0.76 to 13.46 | 0.11 | - | - | - |
| 6-12 months | 8.00 | 1.06 to 60.32 | 0.044 | - | - | - |
| Log Time interval between transplantation and suspicion (+1 day)^e^ | 1.83 | 0.91 to 3.72 | 0.092 | - | - | - |
| Log Treatment duration before suspicion of resistance (+1 day) | 2.24 | 1.03 to 4.87 | 0.042 | 2.24 | 1.03 to 4.87 | 0.042 |

Abbreviations: CI indicates confidence interval; COPD, chronic obstructive pulmonary disease; OR, odds ratio. Data are shown as estimated ORs (95% CIs) of the explanatory variables in the mutations group.

The OR is defined as the probability of membership of the group with mutations divided by the probability of membership of the non-mutations group. The P value is based on the null hypothesis that all ORs relating to an explanatory variable equal unity (no effect).

^a^ The variables analyzed in the univariate analysis were: age, sex, CMV pre-transplant donor/recipient serology (D/R), type of transplant, prophylaxis and induction and maintenance therapy received, number of days after transplantation, if the patient was receiving prophylaxis, preemptive or disease treatment, viral load, CMV disease, and the dose and number of days of GCV or VGCV received prior to the suspicion.

^b^ Hosmer-Lemeshow goodness-of-fit test, p=0.33.

^c^ Predictors from the model can be used to calculate the probability of mutations by the following formula: Exp(β) / (1 + Exp(β)), where β = -3.265 + 0.806 x Log treatment duration before suspicion of resistance.

^d^ The p-value corresponds to differences between the three groups (no, 1-3 months or 3-6 months).

^e^ +1 means a one-unit increase on the scale in the predictor variable (i.e., going from 1 to 2, 2 to 3, etc.).
