# Supplementary material for: Improvement in detecting cytomegalovirus drug resistance mutations in solid organ transplant recipients with suspected resistance using next generation sequencing
Source: PLoS One. 2019 Jul 18;14(7):e0219701. doi: 10.1371/journal.pone.0219701 (PMC6638921; doi:10.1371/journal.pone.0219701)
Supplement: S1 Fig — (DOC) [file pone.0219701.s006.doc]

**S1 Fig. Receiver operating characteristic curve analysis of significant variables derived from the logistic regression model for the ability to predict mutations**

AUC: 0.71

95% CI: 0.53 to 0.88

Abbreviations: AUC indicates area under the curve; CI, confidence interval.
